# CD47 Deficiency Attenuates Isoproterenol-Induced Cardiac Remodeling in Mice

**DOI:** 10.1155/2019/7121763

**Published:** 2019-11-19

**Authors:** Zhi Zuo, Ming-Yue Ji, Kun Zhao, Zhong-Ping Su, Peng Li, Dao-Rong Hou, Yong Li

**Affiliations:** ^1^Department of Cardiovascular, Zhongda Hospital Affiliated to Southeast University, No. 87 Dingjiaqiao, Nanjing, Jiangsu, China; ^2^Department of Cardiovascular, Lianshui People's Hospital, No. 6, East Hongri Avenue, Huaian, Jiangsu, China; ^3^Department of Cardiology, The First Affiliated Hospital of Nanjing Medical University, 300 Guangzhou Road, Nanjing, Jiangsu, China; ^4^Key Laboratory of Model Animal Research, Animal Core Facility of Nanjing Medical University, Nanjing Medical University, 101 Longmian Avenue, Nanjing, Jiangsu, China

## Abstract

In this study, we investigated whether CD47 deficiency attenuates isoproterenol- (ISO-) induced cardiac remodeling in mice. Cardiac remodeling was induced by intraperitoneal (i.p.) injection of ISO (60 mg·kg^−1^·d^−1^ in 100 *μ*l of sterile normal saline) daily for 14 days and was confirmed by increased levels of lactate dehydrogenase (LDH) and creatine kinase MB (CK-MB), increased heart weight to body weight (HW/BW) ratios, and visible cardiac fibrosis. Apoptosis was evaluated by terminal deoxynucleotidyl transferase-mediated dUTP nick end labeling (TUNEL) staining. Levels of malondialdehyde (MDA) and reactive oxygen species (ROS) were found to be significantly higher in the ISO group than in the control group, while superoxide dismutase (SOD) levels were suppressed in the ISO group. However, CD47 knockout significantly limited ISO-induced increases in LDH, CK-MB, and HW/BW ratios, cardiac fibrosis, oxidative stress, and apoptosis in the heart. In addition, CD47 deficiency also increased p-AMPK and LAMP2 expression and decreased HDAC3, cleaved Caspase-3, cleaved Caspase-9, LC3II, and p62 expression in cardiac tissues. In conclusion, CD47 deficiency reduced i.p. ISO-induced cardiac remodeling probably by inhibiting the HDAC3 pathway, improving AMPK signaling and autophagy flux, and rescuing autophagic clearance.

## 1. Introduction

Cardiac remodeling is a major cause of morbidity and mortality worldwide [1]. Epidemiological studies have revealed that cardiac hypertrophy is an independent risk factor for cardiac dysfunction and sudden death [2–4]. Cardiac remodeling occurs as a response of the heart to various stresses; it is characterized by cardiac hypertrophy, cardiac fibrosis, cardiac apoptosis, and heart failure (HF) [5–7] and is regarded as a determinant of the clinical course of heart failure [8]. As patients with major remodeling undergo progressive worsening of cardiac function, slowing or preventing cardiac remodeling has become a new goal of heart failure therapy [9, 10].

*β*-Adrenergic receptors, or *β*-adrenoceptors (*β*-ARs), play important roles in the regulation of cardiac excitation contraction and are essential regulators of cardiovascular homeostasis [11]; however, overactivation of *β*-adrenergic signaling can lead to cardiac remodeling [12, 13]. Treatment with isoproterenol (ISO), a nonselective agonist of *β*-ARs, has been widely used to induce cardiac hypertrophy and subsequent heart failure in experimental animals [14–16]. ISO is the main agent used to induce cardiac hypertrophy models because it is convenient and yields rapid and reproducible results [17]. ISO-induced cardiac injury includes activation of inflammation, necrosis of the myocardium, and disruption of energy reserves in cardiomyocytes; interstitial fibrosis and cardiac remodeling also occur, eventually causing cardiac dysfunction [18, 19]. The pathophysiological and morphological alterations induced by ISO in the heart tissues of experimental animals have also been documented to be similar to those observed in infarcted myocardial tissues of humans [20–23].

CD47 is a widely expressed cell receptor [24] and an activator of nicotinamide adenine dinucleotide phosphate (NADPH) oxidase-mediated reactive oxygen species (ROS) production in vascular cells [25]. Previous work has identified a role for CD47 in limiting blood flow [24] and metabolism [26] and has suggested additional benefits of therapeutic targeting of CD47 in myocardial infarction [27]. CD47 transcript levels have also been reported to have increased in ventricular biopsies from left ventricular heart failure (LVHF) patients [28]. Furthermore, a large number of studies have shown that knockout or inhibition of CD47 profoundly protects cells and normal tissue from death induced by ischemia/reperfusion (I/R) treatment [29–34] and that CD47 deficiency confers prosurvival effects against radiation injury via activation of autophagic flux [35–37]. In vascular cells, CD47 promotes cell injury [38], in part through inhibition of the production and effector pathways of nitric oxide (NO) signaling [39–42] and through pathologic ROS production [43]. Pathologic ROS production plays a role in promoting heart failure [43, 44]. Conversely, mice lacking CD47 are protected from transverse aortic constriction- (TAC-) driven LVHF via enhancing cardiac function and decreased cellular hypertrophy and fibrosis [27]. We hypothesized that CD47 deficiency may have a protective effect on ISO-induced cardiac remodeling. However, thus far, the specific effects of CD47 downregulation on cardiac remodeling induced by ISO have not been reported.

In this study, we used a well-established ISO-induced model of cardiac remodeling to investigate the effects of CD47 deficiency on cardiac remodeling in mice.

## 2. Materials and Methods

### 2.1. Animals

Eighty C57/BL6 male mice, 8-10 weeks of age, and weighing 22-28 g were obtained from Nanjing Medical University. The mice were housed in an SPF facility in the Animal Core Facility of Nanjing Medical University under standard temperature conditions with a 12 h light/dark cycle and were fed Co 60 irradiation-sterilized full-price feed *ad libitum*. All experimental protocols and animal handling procedures were performed according to the “Guide for the Care and Use of Laboratory Animals” (National Academic Press, USA, 1996). The animal study was approved by the Institutional Animal Care and Use Committee of Nanjing Medical University (IACUC-1709018, Sep 13, 2017).

### 2.2. CD47^−/−^ Mice

CD47^−/−^ mice was generated using CRISPR-Cas9 technology. The exon 2 sequence of CD47 gene of C57BL/6 mice was submitted to CRISPR Design Tool (http://crispor.tefor.net/crispor.py). The sgRNAs with high scores were chosen and corresponding oligos were ordered. The specificity of the sgRNA target sites was analyzed according to the basic local alignment search tool (BLAST) applied to the mouse genome. The targeting sequences of the CD47 sgRNA are sgRNA-CD47-1F: TAGGCCCTTGCATCGTCCGTAATG, sgRNA-CD47-1R: AAACCATTACGGACGATGCAAGGG; sgRNA-CD47-2F: TAGGGATAAGCGCGATGCCATGGT, sgRNA-CD47-2R: AAACACCATGGCATCGCGCTTATC. Oligos were synthesized, annealed, and inserted into T7 in vitro transcription vectors (pUC57-sgRNA plasmid, 51132, Addgene). sgRNA plasmids were then digested with Dra I and purified using a MinElute PCR Purification kit (28004, QIAGEN). Transcriptions of the sgRNAs in vitro were performed using a MEGAshortscript kit (AM1354, Ambion). Purifying the sgRNAs was accomplished using a MEGAclear kit (AM1908, Ambion). All of the above operations were according to the manufacturer's instructions. Cas9 mRNA (L-7206) was purchased from TriLink BioTechnologies. Cas9 mRNA and sgRNA were assessed using the Nano-100 spectrophotometer. C57BL/6J mice were superovulated by injection with PMSG (5 IU/100 ml) and HCG (5 IU/100 ml), and zygotes were collected. RNase-free water was used to dilute Cas9 mRNA and sgRNAs to a final concentration of 30 ng/ml. The RNA mixture was microinjected into both the cytoplasm and larger (male) pronucleus of the zygotes. All zygotes were transferred to the pseudopregnant mice within a short time after microinjection. CD47^+/-^ mice interbred with each other to obtain CD47^−/−^ homozygous mice for the following experiments.

### 2.3. Genotyping

The tail tips of mice were boiled in a 300 *μ*l lysis buffer separately. Then, PCR was performed with Taq DNA Polymerase (golden MIX, TsingKe) and with the following primers: CD47-F (5′-GTTACAGTCTACTGGCTGGTGTGCA-3′) and CD47-R (5′-CCCGTGCGGTTTTTCAGCTCTATAA-3′). PCR genotyping yields a 490 bp fragment as expected if deletion happens. Furthermore, the amplified products were cloned into the pMD19T vector for sequencing.

### 2.4. Animal Model of Cardiac Remodeling

An animal model of cardiac remodeling was established by ISO (I5627, Sigma-Aldrich, USA) treatment via intraperitoneal (i.p.) injection (60 mg·kg^−1^·d^−1^, dissolved in sterile normal saline) once daily for 14 consecutive days [45]. After the treatment period, the animals were allowed to recover with free access to food and water. Twenty-four hours after the last administration, the mice were euthanized in a CO_2_ chamber, and the heart tissue was dissected and weighed. The ratio of heart weight to body weight (relative weight of the heart; HW/BW ratio) was measured for each group as an index of cardiac hypertrophy. Blood and left ventricles were harvested for subsequent examination.

### 2.5. Groups and Experimental Protocols

After acclimatization, the animals were randomly divided into the following groups consisting of 20 mice each:

Group 1 *(CD47^+/+^ group)*: the animals in the CD47^+/+^ group received 150 *μ*l of sterile normal saline
Group 2 *(ISO group)*: the CD47^+/+^ animals were injected with ISO (60 mg·kg^−1^·d^−1^ in 100 *μ*l of sterile normal saline, i.p.) daily for 14 days
Group 3 *(CD47^−/−^ group)*: the animals in the CD47^−/−^ group received 150 *μ*l of sterile normal saline
Group 4 *(ISO+CD47^−/−^ group)*: the CD47^−/−^ animals were injected with ISO (60 mg·kg^−1^·d^−1^ in 100 *μ*l of sterile normal saline, i.p.) daily for 14 days

### 2.6. Echocardiography

The systolic heart function of the mice was measured by echocardiography. Briefly, mice were anesthetized by inhalation of 2% isoflurane in the supine position on a heating pad (40°C). Each mouse was allowed to breathe spontaneously, and body temperature was monitored and maintained at 37°C throughout the experiments. Echocardiography was performed with a 35 MHz phased array ultrasound system (Vevo 2100, Visual Sonics Inc., Canada). To minimize data variation, cardiac function was assessed when the heart rate was within the range of 550-650 beats/minute. Data from 3 consecutive heart cycles in each mouse were digitally recorded and analyzed. The following parameters were measured from M-mode images taken from the parasternal short-axis view at the papillary muscle level: interventricular septum thickness (IVS), LV internal dimension (LVID), LV volume (LV vol), LV mass, LV fractional shortening (FS), and LV ejection fraction (EF).

### 2.7. Biochemical Assays

All blood samples were allowed to clot at room temperature and were centrifuged at 2,000 g for 10 min to harvest serum. Biochemical indicators including lactate dehydrogenase (LDH), creatine kinase MB (CK-MB), superoxide dismutase (SOD) and malondialdehyde (MDA) were measured (*n* = 8 mice per group) spectrophotometrically using commercially available kits for LDH (A020-2), CK-MB (H197), SOD (A001-1), and MDA (A003-1) (Jiancheng Bioengineering Institute, Nanjing, China). The HW/BW ratio was calculated by dividing the heart weight (mg) by the body weight (g).

### 2.8. Histological and Morphometric Analysis

The hearts were placed in a 10% potassium chloride solution at end-diastole immediately after removal from the euthanized mice, washed with saline solution, and then placed in 4% paraformaldehyde at 4°C overnight. The hearts were cut transversely close to the apex to visualize the left and right ventricles. The samples were then dehydrated in an ethanol gradient, rinsed in xylene, and embedded in paraffin. Finally, the paraffin blocks were cut into 4 *μ*m sections. The paraffin sections were stained with hematoxylin and eosin (H&E) for histopathology and Masson's trichrome stain for analysis of collagen deposition and were then visualized by light microscopy. The fibrotic area was quantified using Image-Pro Plus.

### 2.9. ROS Staining

Total ROS in fresh frozen sections were stained with dihydroethidium (DHE, D-23107; Invitrogen, USA). Briefly, the hearts taken out of the euthanized mice were mounted in OCT embedding compound (3801480; Leica) and frozen at -80°C. The frozen tissues were cut into 5 *μ*m thick sections using a cryostat and thawed; the sections were then mounted onto gelatin-coated histological slides. Next, 5 *μ*M DHE dissolved in DMSO was added to the fresh frozen mouse heart sections (thickness 5 *μ*m) after dilution in PBS, and the sections were incubated in darkness at 37°C for exactly 30 min. Then, the sections were rinsed twice with cold PBS and immediately imaged.

### 2.10. Q-PCR

Total mRNA was extracted from heart samples using TRIzol reagent (B5704-1, Takara, Dalian, China) and then treated with DNase I (2212, Takara, Dalian, China) according to the manufacturer's protocol. The quality and quantity of RNA were determined using a spectrophotometer (NanoDrop 2000c, Thermo Scientific, USA). cDNA was immediately synthesized using a PrimeScript™ RT Reagent kit (RR037A, Takara, Dalian, China) according to the manufacturer's instructions. Q-PCR was performed using a LightCycler PCR QC kit (Roche, Switzerland) and a 7300 Real-Time PCR System (LC96, Roche, Switzerland). The primer sequences used were as follows: natriuretic peptide precursor type A (ANP) (NM_008725.3) forward: AAGAGGGCAGATCTATCGGA, reverse: TTGGCTTCCAGGCCATAATTG; brain natriuretic peptide (BNP) (NM_001287348.1) forward: TCTTGTGCCCAAAGCAGCTT, reverse: ATGGATCTCCTGAAGGTGCT; beta-myosin heavy chain (*β*-MHC) (NM_001361607.1) forward: TGCAAAGGCTCCAGGTCTGAGGGC, reverse: GCCAACACCAACCTGTCCAAGTTC; and *β*-actin (NM_007393.3) forward: CACGGTTGGCCTTAGGGTTCAG, reverse: GCTGTATTCCCCTCCATCGTG. The housekeeping gene *β*-actin was used as an internal reference. Data analysis was performed, and graphs were produced using GraphPad Prism 5 software.

### 2.11. In Situ TUNEL Staining Assay

A terminal deoxynucleotidyl transferase- (TdT-) mediated deoxyuridine triphosphate (dUTP) nick end labeling (TUNEL) assay was performed according to the manufacturer's instructions (11684817910, Roche, Switzerland). Heart tissues were fixed in 4% paraformaldehyde overnight, dehydrated, embedded in paraffin, sectioned into 4 *μ*m thick sections, and placed on numbered polylysine-coated glass slides. The deparaffinized tissue sections were incubated with proteinase K (20 mg/ml, Sigma-Aldrich, USA) in a humidified chamber for 15 min, and endogenous peroxidase activity was blocked by treating the sections with 3% H_2_O_2_ for 10 min. The sections were then incubated with TdT labeling buffer at 37°C for 1 h in a moist chamber and then counterstained with DAPI. TUNEL-positive cells were stained brown, and nuclei were stained blue. Five random fields per slide (five slides per animal, seven animals per group) were examined. The number of TUNEL-positive cell nuclei (numerator) and the total number of cell nuclei (denominator) were counted as previously described [46]. The percentage of TUNEL-positive cells in each field was analyzed using Image-Pro Plus 6.0 software.

### 2.12. Western Blotting

Western blotting analyses were performed according to previously described methods, with slight modifications (Barrera-Chimal et al., 2015). Briefly, 30 mg of protein was separated by 10% SDS-PAGE and transferred to a nitrocellulose membrane. Each membrane was blocked with 5% nonfat milk in TBST buffer (100 mM NaCl, 10 mM Tris-HCl, pH 7.4, 0.1% Tween-20) for 1 h prior to incubation with primary antibodies against HDAC3 (ab16407; Abcam, USA), adenosine monophosphate-activated protein kinase (AMPK; 2535, Cell Signaling Technology, USA), p-AMPK (5831, Cell Signaling Technology, USA), LC3 (4108, Cell Signaling Technology, USA), Beclin-1 (sc-48341, Santa Cruz Biotechnology, USA), p62 (23214, Cell Signal Technology, USA), LAMP2 (sc-71492, Santa Cruz Biotechnology, USA), cleaved Caspase-3 (9664, Cell Signaling Technology, USA), cleaved Caspase-9 (7237, Cell Signaling Technology, USA), and GAPDH (sc-166574, Santa Cruz Biotechnology, USA) at 4°C overnight followed by incubation with a goat anti-rabbit IgG HRP-conjugated secondary antibody (sc-2004, Santa Cruz Biotechnology) or a goat anti-mouse IgG HRP-conjugated secondary antibody (sc-2005, Santa Cruz Biotechnology). Then, the membranes were washed 3 times in TBST, and the blots were imaged using a ChemiDoc XRS+ Molecular Imager (Bio-Rad) with Pierce ECL Western Blotting Substrate (32209, Thermo Scientific) and analyzed using image analysis software (ImageJ 1.42). The housekeeping protein GAPDH was used as an internal reference. Western blotting quantification was corrected for GAPDH expression prior to normalization.

### 2.13. Statistical Analysis

All assays were independently performed 3 times. All data are presented as the mean ± standard error of the mean and were statistically analyzed using SPSS software, version 13.0. The data were analyzed using one-way ANOVA to determine statistical significance and were further evaluated using Bonferroni post hoc tests. A value of *P* < 0.05 was considered statistically significant.

## 3. Results

### 3.1. Generation of CD47^−/−^ Mice

**Figure 1:**
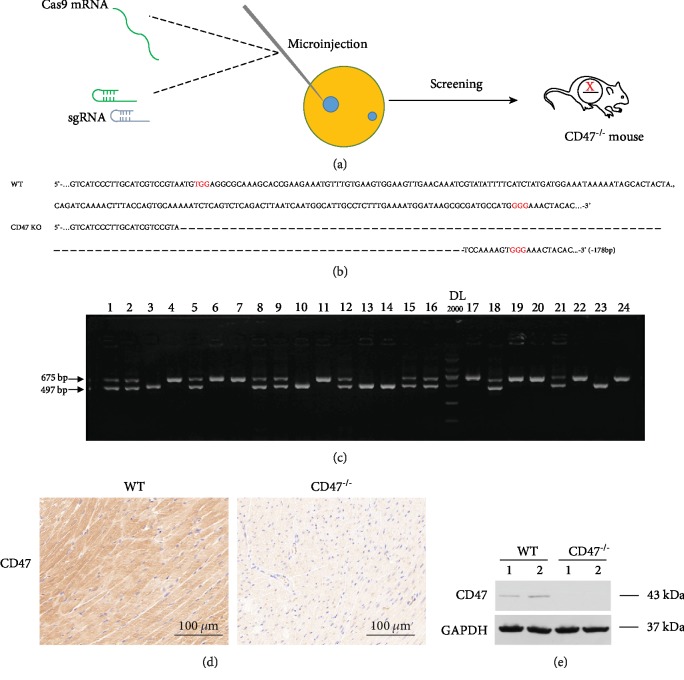
Construction of CD47^−/−^ mice. (a) Construction process of CD47^−/−^ mice generated by CRISPR-Cas9 technology. (b) Gene sequence identification of CD47^−/−^ mice. (c) Representative pictures of immunohistochemical staining of CD47 expression from WT mice and CD47^−/−^ mice. (d) Representative pictures of Western blotting analysis of CD47 expression from WT mice and CD47^−/−^ mice.

To investigate the effect of CD47 deficiency on ISO-induced cardiac remodeling, CD47^−/−^ mice were generated using CRISPR-Cas9 technology (Figure 1(a)). The offspring was genotyped by PCR followed by DNA sequencing analysis. The PCR and DNA sequencing revealed that 178 bp DNA fragment deletions occurred in CD4^7-/-^ mice (Figures 1(b) and 1(c)). Immunohistochemistry and Western blot also showed that there was no protein expression of CD47 in the heart tissue of CD47^−/−^ mice (Figures 1(d) and 1(e)).

### 3.2. CD47 Deficiency Inhibits ISO-Induced Cardiac Remodeling

**Figure 2:**
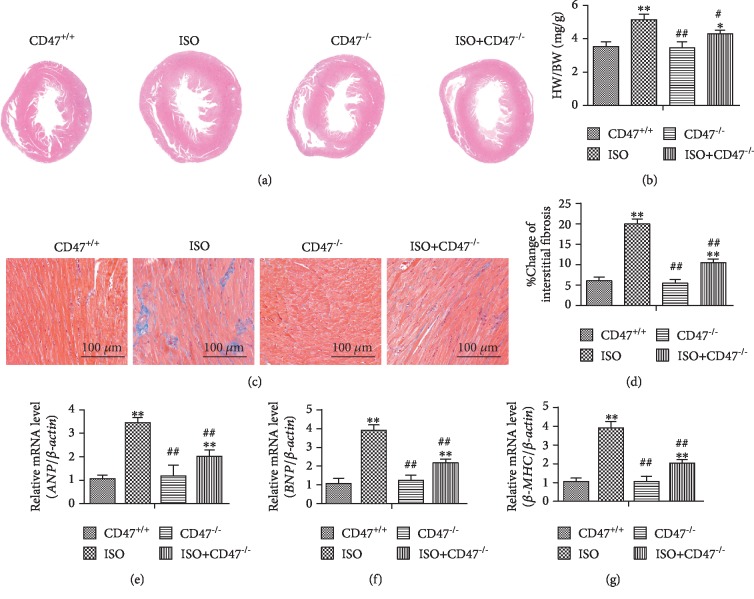
CD47 deficiency inhibits ISO-induced cardiac remodeling. (a) Representative H&E staining of mouse hearts from the four groups. (b) The HW/BW ratios were examined in the four groups (*n* = 8). (c) The levels of fibrosis in the four groups were detected by Masson's trichrome staining. (d) Quantification of the volume of interstitial fibrosis was performed using Image-Pro Plus (*n* = 5). (e) Q-PCR results for hypertrophic gene expression in the four groups (*n* = 8). All data are expressed as the mean ± SD. Statistical significance: ^∗^*P* < 0.05 and ^∗∗^*P* < 0.01 versus the CD47^+/+^ group; ^#^*P* < 0.05 and ^##^*P* < 0.01 versus the ISO group.

No animal death occurred in each group of mice after ISO injection. H&E staining of paraffin sections of hypertrophic hearts showed that the mice injected with ISO had eccentric hypertrophy (Figure 2(a)). HW/BW ratios were significantly higher in mice from the ISO group than in mice from the CD47^+/+^ group (*P* < 0.01, Figure 2(b)). Importantly, the HW/BW ratios of mice from the ISO+CD47^−/−^ group were significantly lower than those of mice from the ISO group (*P* < 0.05).

Cardiac hypertrophy induced by ISO is associated with increased fibrosis in the myocardium [47]. Fibrosis detected by Masson's trichrome staining for collagen was negligible in hearts from mice in the CD47^+/+^ and CD47^−/−^ groups, whereas fibrosis was significantly increased in hearts from mice in the ISO group (*P* < 0.01, Figures 2(c) and 2(d)). Compared with that in mice from the ISO group, fibrosis was significantly lower in mice from the ISO+CD47^−/−^ group (*P* < 0.01).

Pathological hypertrophy and heart failure are also accompanied by altered expression of a large number of genes that correlates with loss of cardiac functions. The hypertrophic genes *β*-MHC, ANP, and BNP are regarded as molecular markers of cardiac hypertrophy [48]. Cardiac expression of the hypertrophy-related genes ANP, BNP, and *β*-MHC was detected by Q-PCR (Figures 2(e)–2(g)). The mRNA levels of ANP, BNP, and *β*-MHC were significantly higher in the ISO group than in the CD47^+/+^ group (*P* < 0.01). The expression levels of ANP, BNP, and *β*-MHC were significantly lower in the ISO+CD47^−/−^ group than those in the ISO group (*P* < 0.01), suggesting an inhibitory effect of CD47 deficiency on hypertrophy-related gene expression.

### 3.3. CD47 Deficiency Attenuates ISO-Induced Cardiac Dysfunction

**Figure 3:**
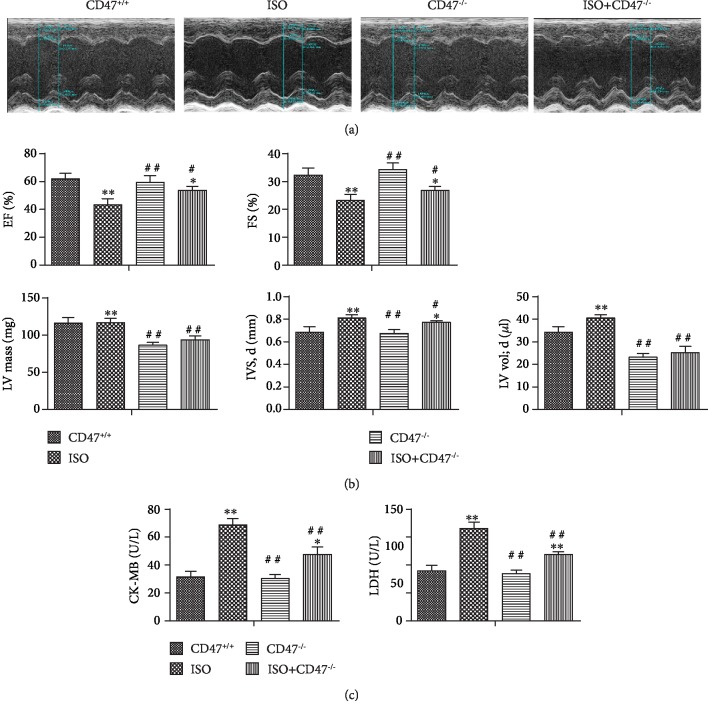
CD47 deficiency attenuates ISO-induced cardiac dysfunction. (a) LV function was measured by echocardiography (*n* = 8). (b) Echocardiography revealed significant impairment in LV function after ISO infusion, as evidenced by changes in EF; FS; LV mass; IVS, d; and LV vol. CD47 antibody treatment improved cardiac function (*n* = 8). (c) Serum CK-MB and LDH levels were measured (*n* = 8). All data are expressed as the mean ± SD. Statistical significance: ^∗^*P* < 0.05 and ^∗∗^*P* < 0.01 versus the CD47^+/+^ group; ^#^*P* < 0.05 and ^##^*P* < 0.01 versus the ISO group.

Echocardiography revealed significant impairment in LV function after ISO infusion, as evidenced by changes in EF and FS (Figure 3(a)). On the other hand, consistent with the H&E results (Figures 3(a) and 3(b)), two consecutive weeks of ISO application caused marked increases in heart size, as evidenced by increases in IVS at end-diastole (IVS, d), LV vol, and LV mass in the ISO group compared with the CD47^+/+^ group (*P* < 0.01, Figure 2(b)), and these physiological changes were prevented by CD47 knockout (*P* < 0.05, *P* < 0.01).

The effects of CD47 deficiency on cardiac function indices are shown in Figures 3(c) and 3(d). There were no significant differences in LDH and CK-MB levels between the CD47^+/+^ and CD47^−/−^ groups, but LDH and CK-MB levels were higher in the ISO group than in the CD47^+/+^ group (*P* < 0.01). CD47 deficiency in the ISO+CD47^−/−^ group decreased LDH and CK-MB levels compared with ISO treatment alone (*P* < 0.01).

### 3.4. CD47 Deficiency Protects against ISO-Induced Oxidative Stress in Cardiac Tissue

**Figure 4:**
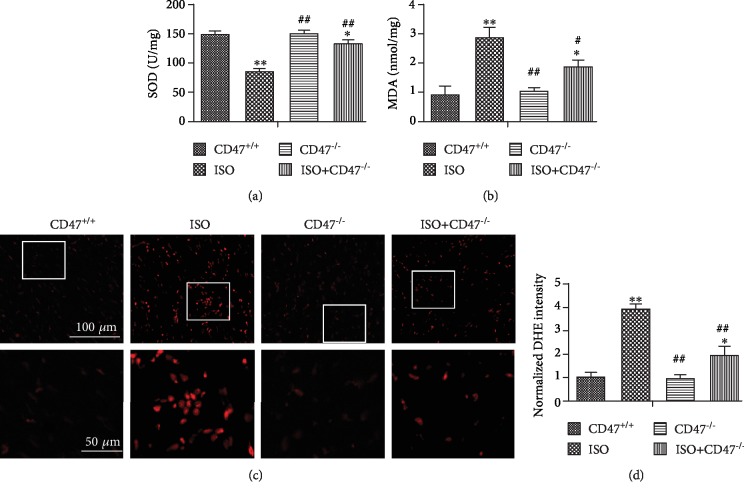
CD47 deficiency protects against ISO-induced oxidative stress in cardiac tissue. The activity of SOD (a) and MDA (b) in serum was measured (*n* = 6). (c) The ROS levels in the hearts of mice from all groups were revealed by DHE staining of frozen sections. (d) The fluorescence intensity of DHE staining was analyzed using Image-Pro Plus (*n* = 6). All data are expressed as the mean ± SD. Statistical significance: ^∗^*P* < 0.05 and ^∗∗^*P* < 0.01 versus the CD47^+/+^ group; ^#^*P* < 0.05 and ^##^*P* < 0.01 versus the ISO group.

The effects of CD47 deficiency on oxidative stress levels in cardiac tissue are shown in Figures 4(a) and 4(b). There were no significant differences in MDA and SOD levels between the CD47^+/+^ and CD47^−/−^ groups, but MDA levels were higher and SOD activity was lower in cardiac tissue from the ISO group than that in the CD47^+/+^ group (*P* < 0.01). CD47 knockout in the ISO+CD47^−/−^ group decreased MDA levels and increased SOD activity compared with ISO alone (*P* < 0.01).

To assess the effects of ISO on ROS production, we visualized intracellular generation of the ROS moiety O^2-^ with the fluoroprobe DHE (Figure 4(c)). The superoxide anion oxidizes DHE to a novel product that binds to DNA, leading to enhanced fluorescence. In this assay, confocal microscopy showed that cardiac tissue sections from the ISO group had widespread and marked increases in DHE fluorescence compared with those from the CD47^+/+^ group (*P* < 0.01, Figure 4(d)), and CD47 knockout in the ISO+CD47^−/−^ group decreased ROS fluorescence intensity compared with ISO alone (*P* < 0.01).

### 3.5. CD47 Deficiency Protects against ISO-Induced Cardiomyocyte Apoptosis

**Figure 5:**
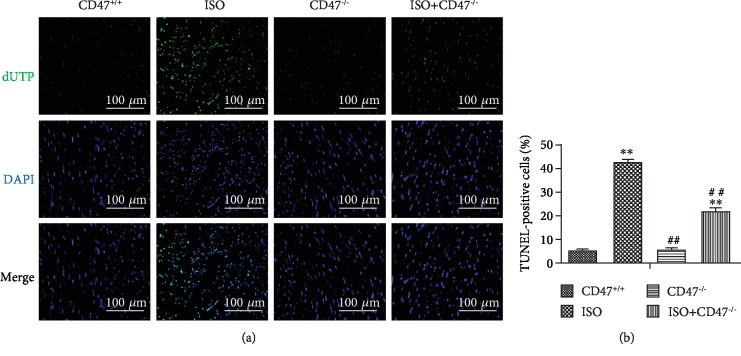
CD47 deficiency protects against ISO-induced cardiomyocyte apoptosis. (a) Apoptosis was analyzed using in situ TUNEL fluorescence staining. The nuclei of TUNEL-positive (apoptotic) cells appeared green. Five random fields per section (five sections per tissue from each mouse) were examined in each experiment. (b) The numbers of TUNEL-positive granulosa cells (%) were compared among the four groups (*n* = 5). All data are expressed as the mean ± SD. Statistical significance: ^∗^*P* < 0.05 and ^∗∗^*P* < 0.01 versus the CD47^+/+^ group; ^#^*P* < 0.05 and ^##^*P* < 0.01 versus the ISO group.

In the TUNEL assay, the nuclei of TUNEL-positive (apoptotic) cells appeared green, indicating apoptotic cells (Figure 5(a)). There were no significant differences in apoptosis rates between the CD47^+/+^ and CD47^−/−^ groups (Figure 4(b)). The levels of apoptosis are indicated as the percentage of TUNEL-positive cells among the total cells (Figure 5(b)). Few apoptotic cells were observed in the CD47^+/+^ and CD47^−/−^ groups, whereas the ISO group displayed more TUNEL-positive cells than the CD47^+/+^ and CD47^−/−^ groups (*P* < 0.01). As expected, CD47 knockout significantly decreased the number of TUNEL-positive cells, and fewer apoptotic cells were observed in the ISO+CD47^−/−^ group than in the ISO group (*P* < 0.01).

### 3.6. Effects of CD47 Deficiency on Cardiac Remodeling-Related Signaling Pathways in Cardiac Tissue

**Figure 6:**
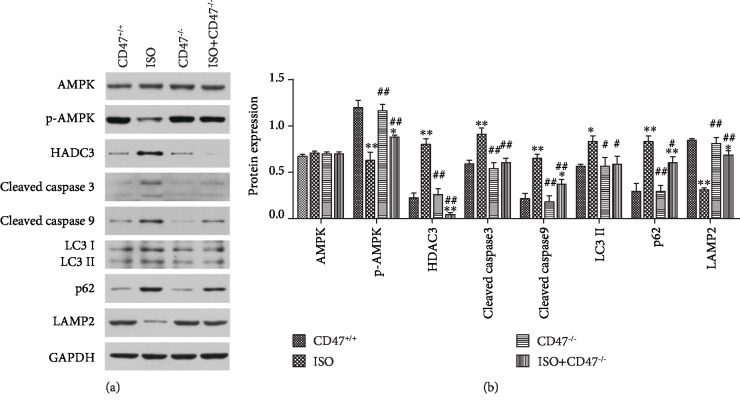
Effects of CD47 deficiency on cardiac remodeling-related signaling pathways. (a) Representative Western blots depicting the protein levels of AMPK, p-AMPK, HDAC3, cleaved Caspase-3, cleaved Caspase-9, LC3 II, Beclin-1, p62, and LAMP2 in heart tissue. (b) The protein expression levels were quantitatively analyzed (*n* = 6). All data are expressed as the mean ± SD. Statistical significance: ^∗^*P* < 0.05 and ^∗∗^*P* < 0.01 versus the CD47^+/+^ group; ^#^*P* < 0.05 and ^##^*P* < 0.01 versus the ISO group.

The potential mechanisms involved in the effects of CD47 deficiency on ISO-induced cardiac remodeling were investigated by Western blot analysis (Figure 6(a)). The expression of total AMPK protein was equal among the groups, while p-AMPK expression was significantly lower in the ISO group than in the CD47^+/+^ group (*P* < 0.01) (Figure 6(b)) and was markedly higher in the ISO+CD47^−/−^ group than in the ISO group (*P* < 0.01). The expression of HDAC3, cleaved Caspase-3, and cleaved Caspase-9 was significantly higher in the ISO group than that in the CD47^+/+^ group (*P* < 0.01). Downregulation of HDAC3, cleaved Caspase-3, and cleaved Caspase-9 was observed in the ISO+CD47^−/−^ group compared with the ISO group (*P* < 0.01).

The present study also investigated the expression of the autophagosomal marker LC3 II, the autophagy-associated proteins Beclin-1 and p62, and the autophagosome clearance-related protein LAMP2 using Western blot analysis. As shown in Figures 6(a) and 6(b), there were no significant differences in the protein expression levels of LC3 II, Beclin-1, p62, and LAMP2 between the CD47^+/+^ and CD47^−/−^ groups. LC3 II, Beclin-1, and p62 protein levels were increased and LAMP2 protein levels were decreased in the ISO group compared with the CD47^+/+^ group (*P* < 0.05 and *P* < 0.01, respectively). CD47 knockout in the ISO+CD47^−/−^ group decreased LC3 II and Beclin-1 and p62 protein levels and increased LAMP2 protein levels compared with ISO alone (*P* < 0.05 and *P* < 0.01, respectively).

## 4. Discussion

Cardiac hypertrophy is a common compensatory response of the heart to acute myocardial injury, infection, or hemodynamic stress [49]. *β*-Adrenergic agonists not only enhance cardiomyocyte proliferation and hypertrophy but also promote cardiac fibroblast proliferation and cardiac dysfunction [50], all of which contribute to cardiac remodeling. The myocardium contains plentiful concentrations of diagnostic markers of myocardial infarction; once metabolically damaged, the myocardium releases these markers into the extracellular fluid [51]. Cytosolic enzymes such as CK-MB and LDH, which serve as diagnostic markers, leak from the damaged tissue into the blood stream when the cell membrane becomes permeable or ruptures [52]. Increases in HW/BW ratios and cardiac fibrosis are also observed in ISO-induced myocardial infarcted mice, indicating cardiac hypertrophy [53]. Such observed increases in heart weight in ISO-induced mice might be due to increases in water content and edematous intramuscular space and to extensive necrosis of cardiac muscle fibers followed by invasion of damaged tissues by inflammatory cells [54]. In the present study, ISO-injected mice showed significant elevations in serum levels of the described marker enzymes. This study also showed that HW/BW ratios and cardiac fibrosis were increased by ISO treatment and that CD47 knockout reversed these changes. We have thus confirmed that CD47 deficiency exerts cardioprotective effects against ISO-induced cardiac remodeling by inhibiting heart failure, cardiac fibrosis, and cardiac hypertrophy.

ROS are important for the development of cardiac hypertrophy [55–57]. Various experimental and clinical studies have shown that enormous amounts of ROS, such as superoxide, hydrogen peroxide, and hydrogen radicals, are generated in failing myocardium [58]. Although low levels of ROS are essential for normal physiological function, excessive ROS produced in dysfunctional mitochondria may compromise and eventually overwhelm the mitochondria, which can result in inflammation, apoptosis, heart dysfunction, and hypertrophy [59–62]. High levels of ROS activate the proapoptotic proteins caspase-3 and caspase-9 [63] and initiate inflammatory responses to I/R injury [59] by increasing the release of inflammatory cytokines [64, 65]. In the present study, ISO administration resulted in marked decreases in SOD and elevations in MDA and ROS levels, consistent with previous reports [51, 58, 66]. CD47 knockout increased the serum levels of SOD while simultaneously decreasing the serum levels of MDA, a lipid peroxidation marker, and levels of ROS. Thus, antioxidant mechanisms are likely some of the pathways by which CD47 deficiency protects myocardial function from disruption induced by ISO.

Apoptosis, also known as programmed cell death, has been shown to be a critical process provoking systolic dysfunction and congestive heart failure [67]. Moreover, clinical studies have demonstrated that adrenergic blockers significantly improve cardiac function and reduce cardiac apoptosis in patients with heart failure [68, 69]. In our study, TUNEL staining revealed that there were marked fewer apoptotic cells in the hearts of ISO-injured CD47^−/−^ mice than in the hearts of ISO-injured CD47^+/+^ mice; the Western blot results also showed downregulation of cleaved Caspase-3 and cleaved Caspase-9 in the CD47 knockout group compared with the ISO group. The molecular mechanisms underlying apoptosis involve caspase cascade activation to promote apoptotic body formation and cell fragmentation to cause cell death [70]. Caspase-3 and Caspase-9 activity is closely coupled to upstream proapoptotic signals [71]; cleaved Caspase-9 and cleaved Caspase-3, the activated forms of the proteins, are involved in a mitochondrion-dependent pathway that eventually leads to apoptosis [72]. Thus, these data suggest that CD47 deficiency could significantly reduce apoptosis via a mitochondrion-dependent pathway in myocardial cells.

AMPK, a serine/threonine kinase and a cell metabolic or redox sensor, plays an important role in cellular control [73, 74]. AMPK signaling appears to have broad implications in cardiovascular diseases such as atherosclerosis, hypertension, myocardial I/R injury, and cardiac remodeling [75–77]. AMPK activation has been shown to attenuate oxidative stress and prevent cardiomyocyte apoptosis [78] and to protect against endothelial dysfunction and atherosclerosis by suppressing oxidative stress and apoptosis [76]. The present study also demonstrated that AMPK activation inhibits, whereas AMPK inhibition promotes, oxidative stress injury and apoptosis in ISO-treated cardiac tissue. AMPK activation by CD47 knockout alleviated cardiac dysfunction and decreased oxidative stress and apoptosis in rats subjected to ISO injection.

HDAC3, which is sensitive to stimulation by ROS and is known to be a proximate activator of tumor necrosis factor alpha [79], plays a role in controlling cardiac responses to stress and has been linked to left heart failure [80]. HDACs also regulate several proteins, including ANP, BNP, *β*-MHC, and a-SKA, to promote myocyte hypertrophy [81, 82], and class I HDACs are being investigated as therapeutic targets in cardiac failure [83, 84]. CD47 is a proximate regulator of HDAC3-driven cellular hypertrophy and ventricular fibrosis. There are multiple mechanisms by which CD47 may upregulate HDAC3 to promote left heart failure [25]. CD47 controls basal [42] and ischemic blood flow [85, 86], and ischemia is known to occur in left heart failure induced by cardiac remodeling [87]. Activation of CD47 may also limit cardiac blood flow under these conditions. Data from the present study revealed that CD47 deficiency attenuated ISO-induced upregulation of HDAC3 in the heart and identified the CD47-HDAC3 axis as an important promoter of cardiac remodeling.

Autophagy plays a beneficial role in the heart in response to pressure overload, *β*-adrenergic stress, and other forms of stress [88]. In particular, autophagy seems to increase protein turnover in the heart undergoing remodeling and to prevent accumulation of abnormal proteins or damaged organelles, which could disrupt cardiac function [89–91]. Emerging evidence indicates that autophagy, an evolutionarily conserved catabolic pathway, is critical in controlling the survival and hypertrophic growth of cardiomyocytes [89]. Loss of autophagy evokes the hypertrophic response and subsequent heart failure, whereas enhancement of autophagy through pharmacological intervention or autophagic gene overexpression can attenuate or even reverse already established cardiac hypertrophy [89, 92–94]. Several molecular mechanisms, including mechanisms involving LC3B II, p62, and LAMP2, are involved in regulation of autophagy [95–98]. In mammalian cells, LC3 II is produced from LC3 I and is modified into a membrane-bound form to prompt its localization to autophagosomes [99]. Thus, LC3 II is considered to be an autophagosomal marker [100]. p62, a protein that links ubiquitinated aggregates for destruction within autophagosomes and is degraded upon autophagosome processing, is upregulated in ISO-treated hearts [101, 102]. The activity of LAMP2, a lysosome membrane protein that plays an important role in autophagosome-lysosome fusion [97, 98], is driven by ROS generation [99]. LAMP2 knockdown impairs autophagy in adult rat ventricular myocytes and causes cell death at levels comparable to those associated with inhibition of autophagy [103], ablation of LAMP2 [89], or loss of LAMP2 [104] protein due to mutations in individuals with Danon disease [105], which is characterized by autophagosome accumulation in multiple tissues, including the myocardium, and by cardiomyopathy [104] and causes extensive myocardial fibrosis [106]. Therefore, the present study examined changes in the levels of these autophagy-associated proteins in the myocardium during cardiac remodeling. The results of this study revealed a rapid decline in LAMP2 abundance and increases in LC3II and p62 abundance in the ISO group and showed that CD47 knockdown improved autophagosome processing by upregulating LAMP2 and downregulating LC3II and p62. It has previously been reported that CD47 deficiency promotes cell survival under conditions of radiation injury through activation of autophagic flux [35–37]. Together, these results suggest that inhibition of autophagy flux-induced autophagosome accumulation is an important pathogenic mechanism for cardiac remodeling. Appropriate activation of cardiomyocyte autophagy may be a novel therapeutic strategy against cardiac hypertrophy and dysfunction.

In conclusion, our findings demonstrate a protective effect of CD47 deficiency against myocardial oxidative stress and apoptosis that is mediated by activation of the AMPK pathway, inhibition of the HDAC3 pathway, improvement of autophagic flux, and rescue of autophagic clearance. Targeting CD47 may be a potential therapeutic approach for preventing cardiac remodeling.

## Data Availability

The data used to support the findings of this study (CD47 deficiency attenuates isoproterenol-induced cardiac remodeling in mice) are available from the corresponding author upon request. Correspondence should be addressed to Daorong Hou (houdaorong@njmu.edu.cn) or Yong Li (liyongmydream@njmu.edu.cn).
